# Improved HaloTag Ligand Enables BRET Imaging With NanoLuc

**DOI:** 10.3389/fchem.2019.00938

**Published:** 2020-01-14

**Authors:** Ovia Margaret Thirukkumaran, Congrong Wang, Nnamdi Joseph Asouzu, Eduard Fron, Susana Rocha, Johan Hofkens, Luke D. Lavis, Hideaki Mizuno

**Affiliations:** ^1^Laboratory for Biomolecular Network Dynamics, Biochemistry, Molecular and Structural Biology Section, Department of Chemistry, KU Leuven, Heverlee, Belgium; ^2^Chem Tech-Molecular Imaging and Photonics, Department of Chemistry, KU Leuven, Heverlee, Belgium; ^3^Howard Hughes Medical Institute, Ashburn, VA, United States

**Keywords:** NanoLuc, HaloTag, BRET imaging, Janelia Fluor dyes, PKA

## Abstract

Bioluminescence resonance energy transfer (BRET) from an exceptionally bright luciferase, NanoLuc, to a fluorescent HaloTag ligand is gaining momentum to monitor molecular interactions. The recommended use of HaloTag618 ligand for the NanoLuc-HaloTag BRET pair is versatile for ensemble experiments due to their well-separated emission bands. However, this system is not applicable for single-cell BRET imaging because of its low BRET efficiency and in turn weak acceptor signals. Here we explored the unprecedented potential of rhodamine based HaloTag ligands, containing azetidine rings, as BRET acceptors. Through a comprehensive evaluation of various commercial and Janelia Fluor HaloTag ligands for improved BRET efficiency and minimal donor signal bleed-through, we identified JF525 to be the best acceptor for microscopic BRET imaging. We successfully employed BRET imaging with JF525 to monitor the interaction of protein kinase A catalytic and regulatory subunit. Single-cell BRET imaging with HaloTag JF525 can henceforth open doors to comprehend and interpret molecular interactions.

## Introduction

Förster resonance energy transfer (FRET) is a process of transferring excited-state energy from one chromophore (donor) to another (acceptor) via non-radiative dipole-dipole coupling. FRET has been a popular choice to detect interaction and conformational change of molecules since FRET depends on distance and relative orientation between the donor and acceptor. The development of a palette of genetically-encoded fluorescent proteins (FPs) has enabled the design of a variety of FRET-based biosensors to monitor intracellular phenomena (Sanford and Palmer, 2017). However, excitation of the donor FP by light illumination, an essential requisite for fluorescence imaging, might induce undesired system perturbation such as cell damage due to phototoxicity, photobleaching, and production of autofluorescence from intrinsic molecules.

Bioluminescence resonance energy transfer (BRET) is a category of FRET with the substitution of a fluorescent donor with a luminescent protein. Since bioluminescence is an intrinsic byproduct of an enzymatic reaction catalyzed by luciferases, BRET eliminates the need for an external excitation light. Employing the previous generation of luciferases (such as Firefly or Renilla luciferases) for imaging requires long exposure time even with a sensitive detection system due to very weak luminescence. Recently a bright luciferase, NanoLuc (England et al., 2016), which is small and emits blue luminescence has been engineered to be a good BRET donor (Machleidt et al., 2015; Hiblot et al., 2017). BRET sensors with NanoLuc and acceptor FP have been developed and applied for live cell imaging (Schaub et al., 2015; Hamer et al., 2017). Besides FPs, self-labeling protein tags such as HaloTag (Los et al., 2008) and SNAP/CLIP-tag (Keppler et al., 2003; Gautier et al., 2008) are alternative acceptors for BRET imaging. These genetically encodable enzyme tags catalyze a covalent bond formation with their fluorescently labeled cognate ligands. Amongst a series of membrane-permeable ligands for live cell assays, HaloTag NanoBRET 618 (Halo618) has been proposed as an appropriate BRET acceptor due to its well-separated emission band that minimizes the impact of signal bleed-through from NanoLuc. The NanoLuc-Halo618 pair has been employed for ensemble BRET experiments (Machleidt et al., 2015; White et al., 2017). However, from a weak absolute acceptor signal employing Halo618, it is difficult to obtain information such as dynamics and heterogeneity from non-synchronized cells via single-cell BRET imaging. The weak signal is due to the limited spectral overlap between NanoLuc emission and Halo618 absorption resulting in a lower BRET efficiency. In contrast, the superior spectral overlap of green-shifted acceptors are expected to improve BRET efficiency although the bleed-through of donor-signal into the acceptor window might become prominent. To enable live cell BRET imaging with NanoLuc, we evaluated rhodol and rhodamine based HaloTag ligands containing azetidine rings [Janelia Fluor 503 (JF503), Janelia Fluor 525 (JF525), Janelia Fluor 549 (JF549)], in comparison to oregon green (OG), tetramethylrhodamine (TMR) and Halo618, in the green-red spectral range for BRET efficiency, acceptor signal intensity and the impact of donor bleed-through.

## Materials and Methods

### Protein Expression and Purification

*E. coli* JM109(DE3) was transformed with the respective bacterial expression vectors encoding either NanoLuc or Halo-NanoLuc. HaloTag was cloned to the C-terminus of NanoLuc with a spacer (SGGS). Transformed bacterial cells were grown, induced with IPTG for protein expression and later harvested to purify the expressed protein. The purified proteins were used for *in vitro* evaluation (details on cloning and protein purification can be found in Supplementary Material).

### Spectral Acquisitions *in vitro*

#### Fluorescence Spectra of HaloTag Ligands

Synthesis of JF503, JF525, JF549, and spectroscopic measurements for HaloTag ligands (Supplementary Figure 2) were performed as described previously (Grimm et al., 2015, 2017). Absorption and fluorescence spectra (except for Halo618) were recorded with Cary Model 100 spectrometer (Varian) and Cary Eclipse fluorometer (Varian), respectively. The absorption spectrum of Halo618 was acquired with NanoDrop 2000 (Thermo Fischer Scientific). Quantum yields were determined using a Quantaurus-QY spectrometer (C11374, Hamamatsu) employing an integrating sphere to measure photons absorbed and emitted by a sample. Reported are average values from triplicates.

#### Luminescence Spectra

Halo-NanoLuc protein was incubated with HaloTag ligands (OG, JF503, JF525, JF549, TMR, and Halo618 5 μM) at ambient temperature for ~4 h to facilitate complete binding. Luminescence spectra for NanoLuc alone or Halo-NanoLuc labeled with the respective ligands were acquired with a final protein concentration of 4 nM in PBS + 0.1% BSA in the presence of furimazine. Luminescence was collected within a few minutes after adding furimazine with a 30-mm lens and sent to a spectrograph (Acton SpectroPro-300i monochromator/spectrograph). The dispersed luminescence from the spectrograph was detected with a sensitive liquid nitrogen cooled CCD camera (Princeton Instruments SPEC 10:100B/LNeXcelon). The acquisition time was set to 5 s, the recorded spectra were averaged 10 times and background subtracted.

### BRET Imaging in Living Cells

Imaging experiments were all performed on transfected cells (protocol in Supplementary Material), within a few minutes after furimazine (1:30 dilution) addition, on an inverted microscope IX83 (Olympus, Tokyo) with an APON 60XOTIRF (NA = 1.49) objective lens (Olympus). Luminescence was split into donor and acceptor windows with a dichroic mirror DM509 (Semrock, NY; OG, JM503, and JM525) or DM555 (Semrock; JF549, TMR, and Halo618). Donor and acceptor images were acquired simultaneously with two EM-CCD cameras (ImagEM C9100-13, Hamamatsu Photonics, Hamamatsu, Japan). The images for Halo-NanoLuc were acquired with 2x binning (0.55 μM per pixel), 1200 EM gain and 0.75 s exposure time (averaged 2x). To observe PKA dissociation events, time traces were recorded for 7 min with 2x binning, 297 EM gain and 1 s exposure time (averaged 2x). After ~3 min, cells were stimulated by adding 10 μM forskolin through a winged infusion set −22G × ¾” (Terumo, Tokyo, Japan). Acceptor/Donor emission ratios (R_A/D_) were calculated from regions of interest (ROI) in individual cells using Fiji (Schindelin et al., 2012). Background from each window was calculated from an ROI without any cells. The background-corrected values were used for further analyses. The different HaloTag ligands were imaged with the same imaging parameters except for the choice of dichroic mirrors as indicated.

## Results

### Green-Shifted HaloTag Ligands Show Better BRET Efficiency

**Figure 1:**
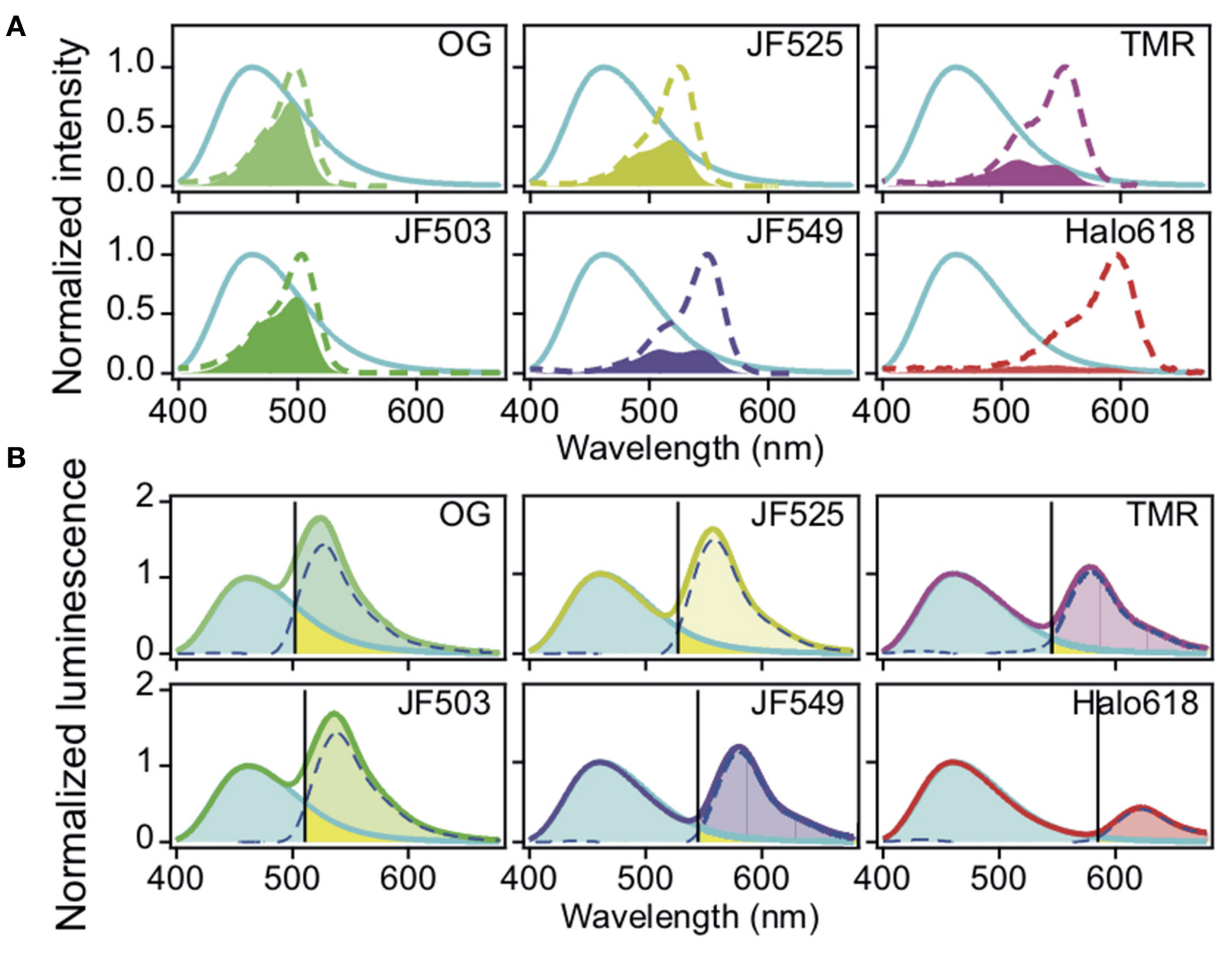
Spectra of NanoLuc and HaloTag ligands. **(A)** Normalized absorption spectra of HaloTag ligands. The spectra of the different ligands are in dotted lines (OG, bluish green, JF503, yellowish green, JM525, yellow, JF549, violet, TMR, purple, Halo618, red) and overlaid with the normalized luminescence spectrum of NanoLuc (cyan solid line). The filled region indicates the overlap area (the products of two spectra). **(B)** Luminescence spectra of Halo-NanoLuc conjugated with Halo-ligands. Spectra were normalized with the donor peak at 460 nm. Dotted blue lines indicate the acceptor spectrum calculated by subtracting the spectrum for only NanoLuc (cyan) from that with the ligand. The wavelength where the acceptor spectrum intersects the NanoLuc spectrum (S_D:A_) is denoted with a black line. R_bt_ is the ratio of yellow area over sum of yellow and cyan areas, whereas F_D_ is the ratio of yellow area over sum of the yellow area and the area colored with the color code of respective ligands.

For the *in vitro* evaluation, we acquired the emission spectrum of recombinant NanoLuc with substrate furimazine and the absorption spectra of different HaloTag ligands: OG, JF503, (Grimm et al., 2017) JF525, (Grimm et al., 2017) JF549, (Grimm et al., 2015), TMR and Halo618 (Figure 1A). Since the efficiency of energy transfer linearly correlates to the overlap integral *(J), J* was calculated using the acquired spectra (Table 1). JF503 had the largest *J* (1.8 × 10^13^ M^−1^ cm^3^) followed by OG and JF525 (83% of JF503), JF549 (72% of JF503), and TMR (56% of JF503). For Halo618, *J* could not be calculated since its extinction coefficient was not available. Assuming Halo618 has an extinction coefficient comparable to the other HaloTag ligands, a *J* smaller than TMR is expected from the overlapped area calculated with the normalized spectra.

**Table 1:** Evaluation of HaloTag ligands as BRET acceptors of NanoLuc *in vitro*^a^.

**Ligand**	**Abs_**max**_** **(nm)**	**ε** **(M**^**−1**^ **cm**^**−1**^**)**	**Em_**max**_** **(nm)**	**Φ**	***J*** **(M^**−1**^ cm^**3**^)**	**I_**A**_**	**S_**D:A**_** **(nm)**	**R_**bt**_** **(%)**	**F_**D**_** **(%)**
OG	496	8.3 × 10^4^	526	0.88	1.5 × 10^13^	86.4	502	25.5	22.1
JF503	503	8.3 × 10^4^	529	0.87	1.8 × 10^13^	90.1	511	19.9	17.2
JF525	525	9.4 × 10^4^	549	0.91	1.5 × 10^13^	82.5	528	12.1	11.9
JF549	549	1.0 × 10^5^	571	0.88	1.3 × 10^13^	63.7	547	6.9	8.8
TMR	554	7.8 × 10^4^	572	0.41	1.0 × 10^13^	65.7	545	7.4	9.8
Halo618	595	ND^b^	621^c^	ND^b^	ND^b^	22.7	585	2.3	8.2

a *Abs_max_, peak of the absorption spectrum; ε, molar extinction coefficient; Em_max_, peak of the emission spectrum; Φ, quantum yield; J, overlap integral; I_A_, bleed-through corrected signal intensity from the acceptor; S_D:A_, the split point between donor and acceptor windows; R_bt_, ratio of NanoLuc signal in the acceptor window to the entire NanoLuc signal; F_D_, fraction of donor signal in the acceptor window relative to the entire acceptor signal. Refer Supplementary Material for calculation of J, R_bt_, and F_D_*.

b *Not determinable due to lack of information on material synthesis*.

c *Data from Promega*.

To experimentally evaluate the BRET efficiency, we acquired the emission spectra of recombinant NanoLuc-HaloTag tandem protein (Halo-NanoLuc) conjugated with the HaloTag ligands (Figure 1B). After normalizing the spectra with the maximum emission of NanoLuc (at 460 nm), signal intensity derived from the acceptor molecule was calculated by subtracting integral of the spectrum for only NanoLuc from that of Halo-NanoLuc conjugated with the ligand (I_A_) (Table 1). Since the spectra were acquired in the dark, energy exciting the acceptor was provided exclusively through BRET, and therefore I_A_ reflects the BRET efficiency as well as the fluorescence quantum yield of the acceptor (Φ). As expected from calculated *J* and Φ, JF503 showed the highest BRET efficiency (*I*_A_ = 90.1) followed by OG (96% of JF503) and JF525 (92% of JF503). I_A_ values of JF549 and TMR were about 70% of JF503 whereas Halo618 was only 25% of JF503. Therefore, we concluded that in terms of BRET efficiency green-shifted ligands performed as better acceptors, which was in accordance with previous reports on non-JF dyes (Machleidt et al., 2015; Hiblot et al., 2017).

### Quantifying Donor Signal Bleed-Through to Identify Best Acceptor

Another crucial factor we evaluated is the donor signal bleed-through. To quantify bleed-through, we determined the wavelength (S_D:A_) at the intersection of NanoLuc and the acceptor spectra (Figure 1B). Based on S_D:A_ value, we calculated the ratio of NanoLuc signal in acceptor window relative to entire NanoLuc signal (R_bt_), and the fraction of signal derived from donor molecules relative to entire signal in the acceptor window (F_D_). R_bt_ gives the degree of bleed-through whereas F_D_ indicates the impact of this bleed-through in the acceptor window. Bleed-through was insignificant for Halo618 (R_bt_ = 2.3%), and it became more prominent for ligands with emission at shorter wavelengths. The R_bt_ values of the ligands in the orange range (JF549, TMR) were about three-fold higher than Halo618. Nevertheless, the impact of bleed through (F_D_) was similar to Halo618 because of a three-fold better acceptor signal (I_A_) (Table 1). On the other hand, ligands in green range (JF525, JF503, and OG) had R_bt_ values ~5–11-fold higher than Halo618. In spite of such large R_bt_ values, the F_D_ values were only 1.5-, 2.1-, 2.7-fold of Halo618 due to brighter acceptor signal (*I*_A_). From this result, we concluded that the impact of bleed-through was not significant compared to the ratio of bleed-through for green-shifted ligands.

### JF525 Performs as an apt Acceptor of NanoLuc

**Figure 2:**
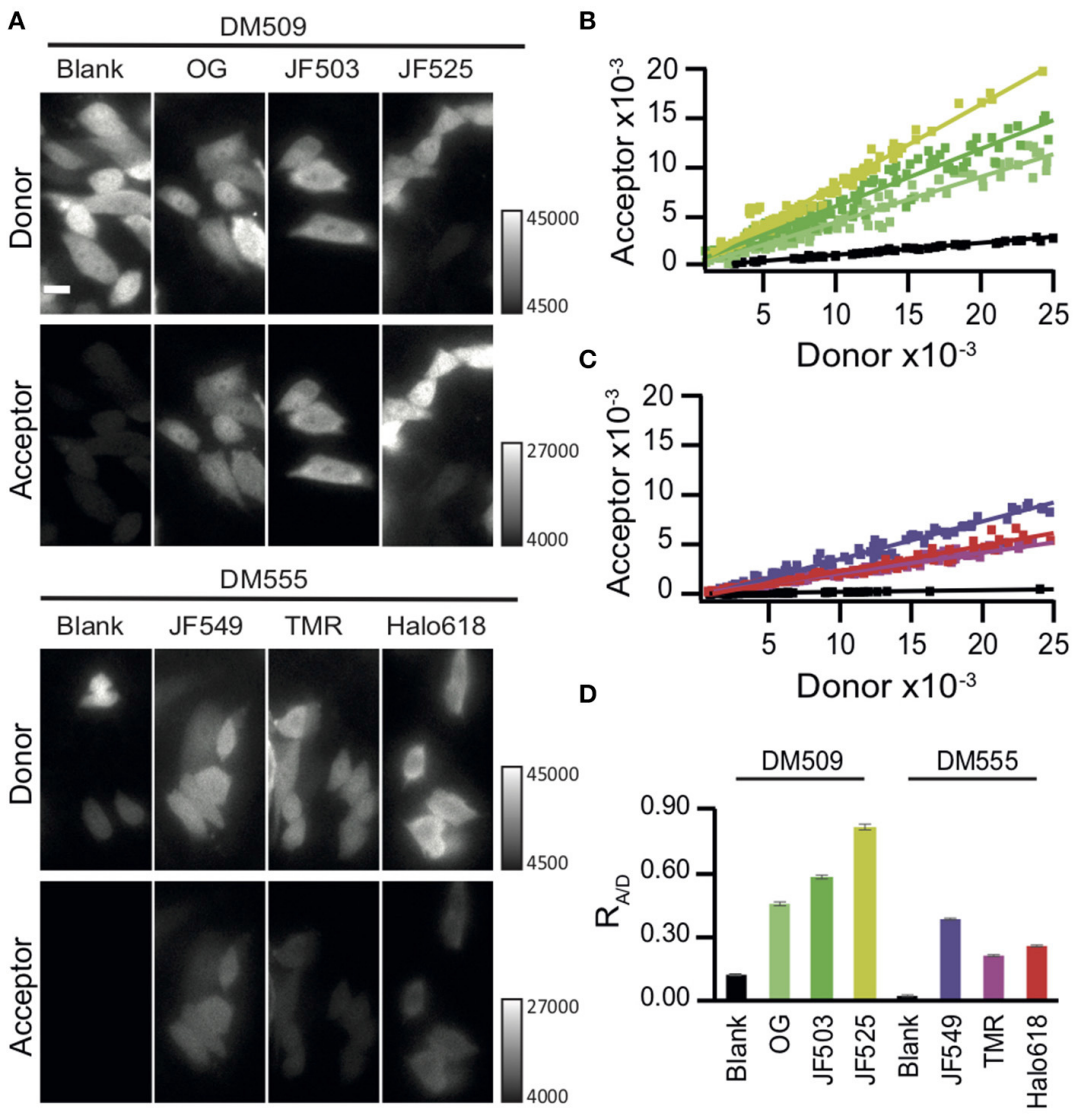
BRET imaging in living cells. **(A)** Images of CHO-K1 cells expressing Halo-NanoLuc. Images were acquired after loading of respective ligands (OG, JF503, JF525, JM549, TMR, and Halo618) or in the absence of ligands (blank), using a dichroic mirror indicated to separate donor (upper) and acceptor (lower) windows. All images were obtained with identical acquisition settings (APON 60XOTIRF, NA = 1.49, exposure time = 0.75 s). Scale bar = 10 μm. **(B,C)** Linear regression analyses of the acceptor vs. donor signal intensities for green-shifted **(B)** or orange-red ligands **(C)**. The color codes of ligand-loaded cells are same as in Figure 1. Cells in the absence of ligands (blank) are shown in black. **(D)** Mean R_A/D_ ± SEM calculated from the cells labeled with different ligands. *n* ≥ 70.

We next evaluated the capability of these different HaloTag ligands for live cell BRET imaging. Chinese hamster ovary (CHO-K1) cells expressing Halo-NanoLuc were labeled with the respective HaloTag ligands and then imaged with a microscope (refer section BRET Imaging in Living Cells for details). The luminescent signals were split into a donor and acceptor window using a dichroic mirror DM509 for the green-shifted ligands (OG, JF503, and JF525) and with DM555 for orange-red ligands (JF549, TMR, and Halo618). Luminescence intensity varies cell by cell depending on the expression level of protein and consumption of furimazine. Therefore, the BRET efficiency was evaluated by comparing the acceptor signal to the donor signal. Bright luminescence signals were observed in a lot of OG-, JF503-, and JF525-loaded cells in both donor and acceptor windows with 0.75 s exposure time, whereas only donor signal was visible in unloaded cells (Figure 2A). In JF549-, TMR-, and Halo618-loaded cells, signals in the acceptor window were barely visible even in cells whose signal in donor window was comparable to OG-, JF503-, and JF525-loaded cells. For quantitative evaluation, signal intensities from acceptor and donor windows were measured for 70 cells or more, and their ratio (R_A/D_) was calculated by performing linear regression analyses (Figures 2B–D). JF525-loaded cells gave the best acceptor signal with the R_A/D_ value of 0.82 ± 0.01 (Mean ± SEM). It was 6.8-fold larger than the bleed-through signal measured in cells without ligands using the same experimental setup (R_A/D_ = 0.12 ± 0.00). JF503 (R_A/D_ = 0.58 ± 0.01) and OG (R_A/D_ = 0.45 ± 0.00)-loaded cells showed signals that were 4.8- and 3.8-fold more than bleed-through. For JF549-, TMR-, and Halo618-loaded cells, the bleed-through was lower (R_A/D_ = 0.02 ± 0.00) as a result of using DM555 instead of DM509, but the acceptor signal was remarkably weaker (R_A/D_ = 0.36 ± 0.00, 0.21 ± 0.00, and 0.24 ± 0.00, respectively) than JF525-loaded cells. We concluded that JF525 was the best acceptor for BRET imaging with NanoLuc.

### Monitoring PKA Activation Through BRET Imaging

**Figure 3:**
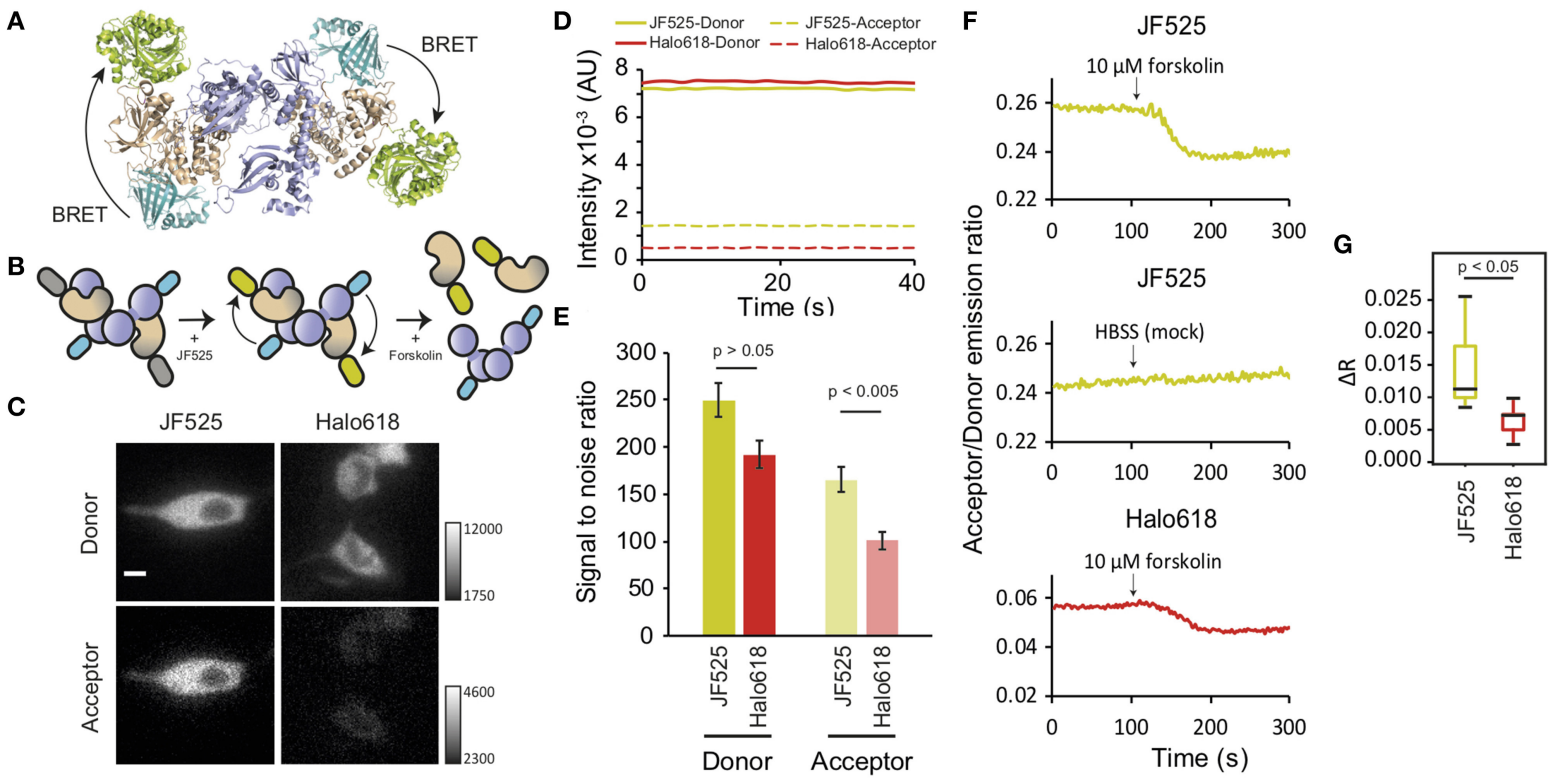
Dissociation of PKA subunits observed by BRET. **(A)** Interaction of NL-RS and CS-HT modeled with the crystal structures (PDB files: 2QBVS, 2F7E, 5IBO, 5Y2Y). The regulatory subunit is shown in purple, catalytic subunit in wheat, NanoLuc in cyan, and HaloTag in greenish-yellow. **(B)** Schematic explaining the decrease in BRET upon dissociation of PKA subunits. The tetrameric structure of tagged NL-RS and CS-HT under resting condition (left) is labeled with HaloTag ligands (middle) and stimulated with forskolin to dissociate the interaction of RS and CS (right). **(C)** Luminescence images of NIH3T3 cells co-expressing NL-RS and CS-HT. Images were acquired after loading respective HaloTag ligands (JF525 and Halo618). All images were acquired with identical camera settings. Scale bar = 10 μm. **(D)** Representative intensity traces of donor and acceptor signals and **(E)** Mean SNR ± SEM for cells labeled with either JF525 or Halo618. **(F)** Time traces of acceptor/donor emission ratios showing dissociation of PKA subunits NL-RS and CS-HT upon forskolin stimulation (indicated by black arrow). **(G)** Box plot showing ΔR_A/D_ values for cells labeled with respective HaloTag ligands. *n* = 8.

Resonance energy transfer systems are frequently employed to monitor protein interactions. Therefore, we evaluated the suitability of BRET imaging with NanoLuc and JF525 for this application. We observed the interaction of regulatory and catalytic subunits of protein kinase A (PRKAR2A and PRKACA, respectively), as these subunits exist together as tetramers under resting conditions (Figure 3A) and undergo a rapid dissociation in response to increase in cAMP levels (Figure 3B) (Taylor et al., 1990; Knighton et al., 1991). We labeled the N-terminus of regulatory (RS)- and C-terminus of catalytic (CS)-subunits with NanoLuc (NL) and HaloTag (HT), respectively (NL-RS, CS-HT). Both the subunits were co-expressed in NIH3T3 cells, labeled with HaloTag ligands and were imaged in the presence of furimazine. Signals were visible in both donor and acceptor windows for cells loaded with JF525 (Figure 3C). From the intensity time trace of the donor and acceptor signal from multiple cells (*n* = 8), the signal to noise ratios (SNRs) for JF525 were determined to be 249.7 ± 18 (donor) and 165.5 ± 14.2 (acceptor) (Figures 3D,E and Supplementary Figure 1). On the contrary, only a faint acceptor signal was observed with Halo618 even for cells showing similar donor intensities as JF525. The difference in donor SNRs was insignificant between JF525 and Halo618 (191.9 ± 13.4; *p* > 0.05) while the acceptor SNR for Halo618 (100.9 ± 8.9) was significantly lower (*p* < 0.005). The dissociation of PKA subunits was evident through a decrease in R_A/D_ upon forskolin, an agonist for adenylyl cyclase to increase cAMP level, addition for cells loaded with JF525 and Halo618 (Figure 3F). However, the response (ΔR_A/D_) for JF525 was 0.012 ± 0.006 (median ± SD), which is twice as much as ΔR_A/D_ for Halo618 (0.007 ± 0.002; *p* < 0.05) (Figure 3G). JF525 evidently showed much brighter acceptor signals suitable to monitor protein interactions by BRET imaging with single-cell resolution.

## Discussion

Self-labeling protein tags such as HaloTag provide a sophisticated platform to evaluate the acceptors in an individual BRET system due to the availability of versatile bright, photostable, and cell permeable ligands. Usage of HaloTag618 as a recommended acceptor for NanoLuc minimizes donor signal bleed-through in the acceptor window (Machleidt et al., 2015). However, a very low energy transfer efficiency yields poor acceptor signal largely hampering its application in the direction of cellular imaging. Employing ligands in the green spectral region, such as Alexa 488, have been reported to show higher BRET efficiency (Machleidt et al., 2015; Hiblot et al., 2017). Since higher bleed-through was expected with green ligands, they were not popularized for ensemble experiments. Conversely, higher absolute acceptor signals are appreciated for BRET imaging. Therefore, we performed a systematic evaluation of a series of HaloTag ligands to identify the best BRET acceptors of NanoLuc for live cell imaging. Brightness comparison amongst the ligands in the green-yellow spectral range (OG, JG503, and JF525) showed JF525 to be better because of a higher extinction coefficient and quantum yield although the overlap integral was lower than JF503. On the other hand, a comprehensive analysis indicated bleed-through to be 1.5-fold lower than OG. With an extensive evaluation of the bleed-through relative to BRET efficiency, we concluded that JF525 was the best *in vitro* amongst the other ligands. Endorsing the *in vitro* results, from cellular BRET imaging it was evident that JF525 showed the highest BRET ratio.

We translated the application of this BRET system with JF525 to monitor protein-protein interaction of the labeled catalytic and regulatory subunits of PKA. Under resting conditions, we observed a basal BRET ratio which showed the interaction of these subunits. Upon stimulation with forskolin, a drop in BRET ratio was detected due to the dissociation of regulatory and catalytic subunits which enabled us to monitor PKA activation. The observed contrast upon stimulation allowed us to monitor dynamic protein-interaction within the sensor system. Apart from monitoring protein-protein interactions, the application of this system could be extended to image the interaction of proteins with small molecules that can be labeled with JF dyes. Moreover, BRET imaging could be interesting to observe interactions on light sensitive cells, avoid phototoxicity and cellular autofluorescence associated with excitation.

In addition to the photochemical properties, cellular permeability and labeling efficiency are important for live cell imaging. Since the Oregon Green fluorophore (2′,7′-difluorofluorescein) has two OH groups at the 3,9′ positions of the xanthene ring (Sun et al., 1997), the fluorophore itself is hydrophilic and cell impermeable. The OG HaloTag ligand is permeabilzed by diacetylating these two OH groups (Supplementary Figure 2). However, the ester bonds are chemically less stable in aqueous solution (Lavis et al., 2011) and its deacetylation decreases the propensity of OG to be cell permeable. Throughout our experiments, fresh stocks of OG were used to avoid this bias. In the case of JF ligands, the presence of azetidine group at the positions 3,9′ of the xanthene ring makes them hydrophobic, facilitating cell permeability. Hence, the stability of JF ligands is not expected to influence the BRET efficiency.

In order to achieve complete labeling in living cells, binding of OG, TMR, and Halo618 was done according to the protocol recommended by Promega. For the JF ligands, we established a protocol based on the reported time course of HistoneH2B labeling with JF HaloTag ligands (Grimm et al., 2017). Under these conditions, a complete labeling is expected to identify the best ligands. Ligands unable to achieve this labeling efficiency introduce underestimation of their BRET efficiencies and such ligands are in any case not appropriate as BRET acceptors.

In conclusion, we have demonstrated that the brightness, cellular permeability, labeling efficiency, photostability of HaloTag ligands, and impact of bleed-through signals relative to acceptor signals are crucial factors for BRET efficiency. Analyses *in vitro* revealed that green-shifted ligands outperformed the red-shifted ligands in terms of better BRET efficiency without marked interference from bleed-through of donor signal. Among the green-shifted ligands, JF525 was identified as the best acceptor for BRET imaging. Bright acceptor images could be acquired from single-cells employing JF525 with an exposure time of <1 s. BRET imaging with JF525 serves as an improved platform to investigate dynamic molecular interactions in single-cells.

## Data Availability Statement

All datasets from this study are included in the manuscript and the Supplementary Files. The raw data files would be available upon request to the corresponding author.

## Author Contributions

OT and HM conceptualized, designed the experiments and wrote the manuscript. OT, HM, EF, CW, and NA acquired the absorption spectra and the luminescence spectra, and performed statistical analyses. LL synthesized and carried out the *in vitro* evaluation of Janelia Fluor (JF) ligands. JH and SR imparted microscopic knowledge and adapted the microscope for bioluminescence imaging. JH, SR, LL, and HM improvised, revised, and approved the submitted version of manuscript.

### Conflict of Interest

The authors declare that the research was conducted in the absence of any commercial or financial relationships that could be construed as a potential conflict of interest.
