# Supplementary material for: Improved HaloTag Ligand Enables BRET Imaging With NanoLuc
Source: Front Chem. 2020 Jan 14;7:938. doi: 10.3389/fchem.2019.00938 (PMC6970966; doi:10.3389/fchem.2019.00938)
Supplement: Supplementary file 1 [file Table_1.docx]

Supplementary Material

# Supplementary Methods

## Materials

The genes encoding NanoLuc, HaloTag, PKA regulatory (PRKAR2A) and catalytic subunits (PRKACA), the HaloTag ligands (Oregon green, TMR, and NanoBRET 618), and furimazine were from Promega (WI, USA). Dulbecco’s modified eagle medium (DMEM), Hank’s balanced salt solution without phenol red (HBSS), fetal bovine serum (FBS), Gentamycin, Trypsin, Dulbecco’s phosphate buffered saline (PBS) were purchased from Thermo Fischer Scientific (MA, USA). Ham’s F-12 medium, XtremeGENE9 and the chemicals used for protein purification were from Sigma-Aldrich (MO, USA) unless specified.

## Molecular cloning

NanoLuc was subcloned into pRSETb at XhoI/EcoRI sites and a tandem of NanoLuc and HaloTag (Halo-NanoLuc) was encoded into NcoI/XhoI sites of pET28a for bacterial expression. Halo-NanoLuc was fused by introducing a BamHI restriction enzyme site (in frame) to the C-terminus of NanoLuc and N-terminus of HaloTag. Additionally, a linker (SGGS) was introduced in the fusion construct between BamHI and HaloTag to minimize steric hindrance. For mammalian expression, Halo-NanoLuc and the labeled PKA subunits were cloned into pcDNA3 at HindIII/XbaI sites. The PKA regulatory subunit was labeled at the N-terminus with NanoLuc (NL-RS) while the catalytic subunit was labeled with HaloTag at C-terminus (CS-HT). The labels and the subunits were linked together with a BamHI restriction enzyme site and an additional GG-linker. All the different plasmids were constructed with DNA fragments amplified using PCR with respective primers, followed by standard restriction enzyme cloning method. The purified plasmids were verified by sequencing.

## Protein expression and purification

100 ml cultures (LB) of transformed bacteria were prepared and incubated at 20ºC overnight to reach an optimal optical density (OD @ 600 nm) of approximately 0.4. Protein expression was induced by adding IPTG (100 µM final) and the cells were incubated further. After 24h, cells were harvested and then re-suspended in 2 ml PBS containing protease inhibitor cocktail and lysozyme (0.2 mg ml^-1^ final). Cells were frozen in liquid nitrogen and thawed sequentially for 3-4 cycles. DNase (0.05 mg ml^-1^ final) and MgCl_2_ (1 mM) were added after the final thawing step and were further incubated on ice for 30 min. Thereafter, the suspension was clarified by centrifugation (15,000×*g* for 10 min at 4ºC). The proteins of interest containing 6x-His tag were captured from the supernatant using Ni-NTA agarose (Qiagen, Germany) resin and eluted with Tris buffer (50 mM Tris/HCl pH7.5, 300 mM NaCl) containing 100 mM imidazole. The purified proteins were used for in vitro evaluation.

## Cell culture

Chinese Hamster ovary cells, CHO-K1, and mouse embryonic fibroblast cells, NIH3T3, were maintained in Ham's F-12 Nutrient Mixture and DMEM at 37°C under 5% CO_2_ atmosphere, respectively. Both Ham’s F-12 and DMEM were supplemented with 10% FBS and 50 μg ml^-1^ Gentamycin. Two days prior to imaging, cells were trypsinized, adjusted to 1.5 x 10^5^ cells per ml, transfected with XtremeGENE9 with the recommended protocol, and 300 µl of the cell suspension were seeded onto 29 mm glass-bottom cell culture dishes (Cellvis, CA, USA). Before imaging, cells were incubated in fresh media containing each of the different HaloTag ligands (OG, JF503, JF525, JF549, TMR, and Halo618; 500 nM) for approximately 3 h to facilitate complete labeling. Cells were washed thrice with HBSS to remove the unbound ligands and were imaged in HBSS+0.2% BSA in the presence of furimazine (recommended dilution). Cells were transfected with either Halo-NanoLuc plasmid (CHO-K1 cells) or with plasmids encoding NL-RS and CS-HT (NIH3T3 cells), and subjected to imaging.

## Calculations, data presentation and analytical software

All analyses were carried out using Excel Office 365 ProPlus (Microsoft, WA, USA), Igor Pro 8 (Wavemetrics, OR, USA), Fiji (Schindelin et al., 2012) and/or Python 3.7 (Anaconda enterprise, TX, USA). Figures were made with Adobe Illustrator CS6 (Adobe Systems, CA, USA).

Overlap integral $(J)$ was calculated with the equation (Lakowicz, 2006):

$$J=\frac{\int_{0}^{\infty} F_{D}\left( \lambda\right)\varepsilon_{A}\left( \lambda\right)\lambda^{4}d\lambda}{\int_{0}^{\infty} F_{D}\left( \lambda\right)d\lambda}$$

where $F_{D}\left( \lambda\right)$ is the emission of NanoLuc, $\varepsilon_{A}\left( \lambda\right)$ is the extinction coefficient of the acceptor in M^-1^cm^-1^, and $\lambda$ is the wavelength in cm.

The ratio (R_bt_) and the fraction of bleed-through (F_D_) were calculated using the following formulae:

$R_{bt}=\frac{I_{DinA}}{I_{D}}$

$F_{D}=\frac{I_{DinA}}{I_{DinA}+I_{AinA}}$

where I_DinA_ is donor bleed-through intensity in the acceptor window, I_D_ is the entire donor intensity, I_AinA_ is acceptor intensity in the acceptor window.

**Steps to calculate the values mentioned in Table 1:**

Step 1: Acquisition of NanoLuc emission spectra

Step 2: Acquisition of NanoLuc + HaloTag (+OG, as an example) emission spectra and normalisation to NanoLuc emission.

Step 3: Calculation of donor bleed-through corrected acceptor BRET signals (I_A_) was executed by subtracting the donor emission (NanoLuc only) from the emission of NanoLuc-HaloTag tandem labelled with respective HaloTag ligands

-

=

Step 4: Calculation of ratio of NanoLuc signal in acceptor window relative to the entire NanoLuc signal (R_bt_)

- The wavelength (vertical black line) at which the emission of NanoLuc intersects with the emission of bleed-through corrected respective acceptor signals was identified by overlaying the emission spectra.

- R_bt_ was calculated by considering the fraction of donor signals observed in acceptor window relative to the donor signals in donor and acceptor window with the equation mentioned above, where I_DinA_ is the Donor signal in acceptor window (region shaded in blue after the black line) and I_D_ is the donor signal in donor + acceptor window (region shaded in cyan before the black line+region shaded in blue).

Step 5: Calculation of fraction of the donor signal in the acceptor window relative to the entire acceptor signal (F_D_). F_D_ was calculated as mentioned in the formula above. I_AinA_ is given in mild yellow, I_DinA_ is highlighted in blue after the intersection wavelength.

**Significance test** for linear regression lines (figure 3E, supplementary figure 1) was calculated with the following equations (Howell, 2009):

- $S_{b1-b2}=\sqrt{{S_{b1}}^{2}+ {S_{b2}}^{2}}$
- $t=\frac{\left( b1-b2 \right)}{\sqrt{{S_{b1}}^{2}+{S_{b2}}^{2}}}$
- ${S_{Res}}^{2}= \frac{\left( n_{1}-2 \right){S_{Res1}}^{2}+(n_{2}-2){S_{Res2}}^{2}}{\left( n_{1}-2 \right)+(n_{2}-2)}$
- ${S_{b1}}^{2}=\frac{{S_{Res1}}^{2}}{{S_{x1}}^{2}(n_{1}-1)}$ ${S_{b2}}^{2}=\frac{{S_{Res2}}^{2}}{{S_{x2}}^{2}(n_{2}-1)}$
- $S_{b1-b2}=S_{Res}\sqrt{\frac{1}{{S_{x1}}^{2}\left( n_{1}-1 \right)}+\frac{1}{{S_{x2}}^{2}\left( n_{2}-1 \right)}}$

where *S_b_* is the standard error of b, *t* is the ratio distribution, *S_Res1_^2^* and *S_Res2_^2^* are the error variances for the two samples, *n* is the number of samples, *S_x_* is the standard deviation of x. p-value was calculated by applying a two-tailed distribution around *t*.

# Supplementary Figures

#
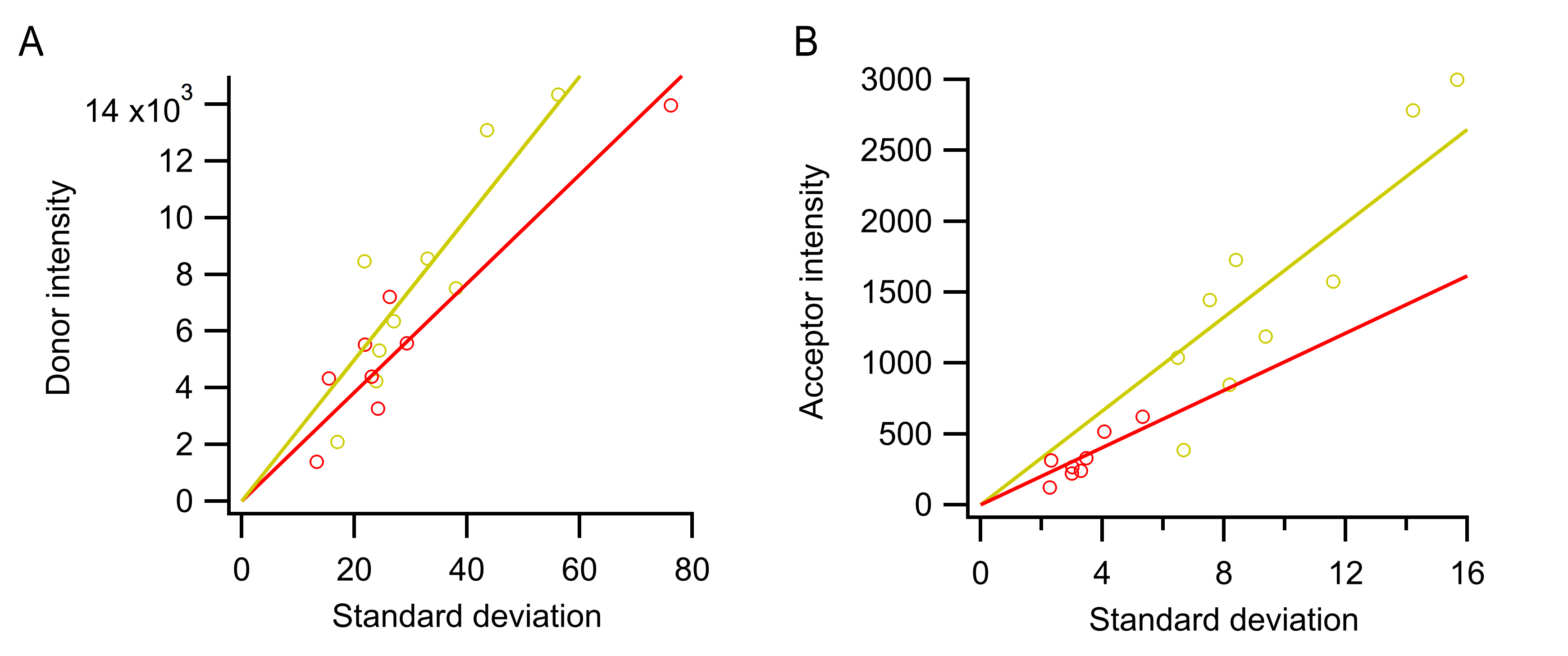


# Supplementary Figure 1. Signal to noise ratio calculation. Mean intensities vs standard deviation of the donor (A) and the acceptor (B) from individual cells. The slope from the linear regression lines gives the signal to noise ratio. The intensity traces for JF525 are in yellow and in red for Halo618. n=8.


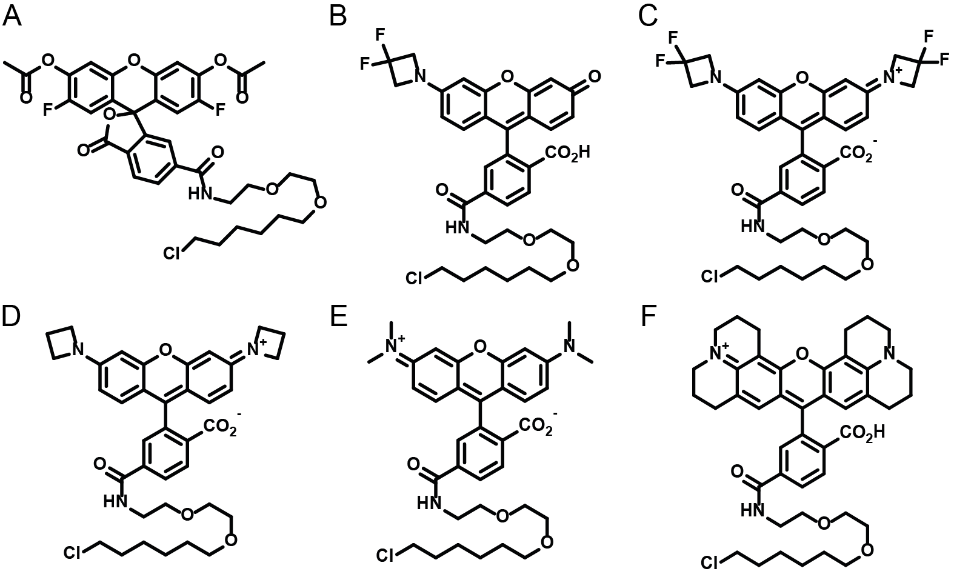


**Supplementary Figure 2.** Structure of HaloTag ligands. Chemical structures of diacetoxy-OG (cell permeable) (A), JF503 (B), JF525 (C), JF549 (D), TMR (E) and Halo618 (F) HaloTag ligands.

# References

Howell, D. C. (2009). *Statistical Methods for Psychology*. p273.

Lakowicz, J. R. (2006). *Principles of fluorescence spectroscopy*.

Schindelin, J., Arganda-Carreras, I., Frise, E., Kaynig, V., Longair, M., Pietzsch, T., Preibisch, S., Rueden, C., Saalfeld, S., Schmid, B., Tinevez, J. Y., White, D. J., Hartenstein, V., Eliceiri, K., Tomancak, P., Cardona, A. (2012). Fiji: an open-source platform for biological-image analysis. *Nature methods*, 9, 676–682.
